# The Expanding Role of Artificial Intelligence in Companion Animal Care: A Systematic Review

**DOI:** 10.3390/ani16071035

**Published:** 2026-03-28

**Authors:** Ivana Sabolek, Alan Jović

**Affiliations:** 1 University of Zagreb Faculty of Veterinary Medicine, Heinzelova 55, 10000 Zagreb, Croatia; 2 University of Zagreb Faculty of Electrical Engineering and Computing, Unska 3, 10000 Zagreb, Croatia; alan.jovic@fer.unizg.hr

**Keywords:** companion animals, artificial intelligence (AI), clinical diagnostics, behaviour, welfare

## Abstract

The rapid increase in companion animal ownership has intensified the demand for innovative tools that support animal health and overall welfare. In recent years, artificial intelligence (AI), particularly machine learning (ML) and deep learning (DL), has emerged as a promising approach in veterinary medicine. However, its application beyond clinical diagnostics, especially in behaviour and personality assessment, remains fragmented and insufficiently integrated into routine practice. This systematic review aims to synthesise current knowledge on AI-based applications in companion animal care, with a focus on behavioural monitoring, personality prediction, and welfare-related challenges. Following PRISMA guidelines, a structured literature search was conducted in the Scopus and PubMed databases from 2020 to 2025. In addition, grey literature sources were searched to capture relevant non-peer-reviewed data. A total of 115 studies met the inclusion criteria and were included in the analysis. Eligibility criteria included studies applying AI methods (machine learning or deep learning) to companion animals (dogs, cats, and exotic pets), while studies on humans, farm animals, or without AI methods were excluded. Due to the heterogeneity of included studies, no formal risk of bias assessment was performed, and results were synthesised narratively. The findings indicate that AI applications are most advanced in diagnostic imaging and clinical decision support, where data availability and methodological maturity are highest. In contrast, AI-based approaches for behaviour and personality prediction remain limited, particularly in cats and exotic companion animals, largely due to small, heterogeneous datasets, potential bias, and a lack of external validation. Emerging technologies such as wearable sensors, computer vision, and multimodal data integration demonstrate substantial potential for continuous behavioural monitoring and early detection of welfare-related issues in real household environments. Nevertheless, significant challenges persist, including data heterogeneity, limited model explainability, ethical considerations, and the absence of regulatory frameworks specifically addressing AI-based veterinary applications. Overall, this review highlights a substantial gap between the technical potential of AI and its current readiness for widespread application in companion animal behaviour and welfare assessment. Future research should prioritise large-scale and standardised data collection, cross-species validation, and interdisciplinary collaboration to ensure that AI-driven tools effectively support veterinary decision-making, animal welfare, and the well-being of owners.

## Simple Summary

Companion animals are increasingly seen as family members, making their health and welfare a growing priority. Artificial intelligence (AI) is being introduced into veterinary medicine, mainly to support disease diagnosis using medical images and clinical data. However, its use in monitoring behaviour, assessing personality, and identifying early welfare problems in home environments of companion animals is still limited. This review summarises recent research on AI applications in dogs, cats, and exotic pets. While AI performs well in diagnostic imaging, studies on behaviour and personality remain scarce, especially for cats and non-traditional companion animals. New tools such as wearable sensors and video analysis show promise for continuous welfare monitoring, but important challenges remain, including limited data, validation issues, and ethical concerns. AI has strong potential to support animal welfare, but further research is needed for its responsible and effective use.

## 1. Introduction

Companion animal ownership has been steadily increasing worldwide. In Europe alone, more than 106 million dogs and 129 million cats were reported in 2024, illustrating a societal shift in which cohabitation with companion animals has become the norm rather than the exception [1,2]. For owners, preservation of the health and welfare of their animals is a primary concern, which underscores the importance of access to accurate and reliable information for informed decision-making [3].

Artificial intelligence (AI) represents a field of computer science focused on developing systems capable of carrying out tasks that simulate aspects of human intelligence [4] and has become embedded across multiple domains of human life [5]. Recent advances have also extended to the field of companion animal care, particularly for dogs and cats, where AI applications range from diagnostics and health monitoring to assessing behaviour and personality traits [2,6].

Current AI development largely focuses on machine learning (ML), which involves systems that can automatically learn useful patterns from data. Machine learning systems rely on statistical algorithms that analyze the available data and build an internal representation (i.e., a model) that enables them to generalize to new, unseen data. Such models can uncover important patterns that may not be easily noticeable to humans [7]. Two major paradigms of ML relevant to companion animal care applications are: (1) supervised learning, where each training example has a known label (class or value) and the goal is to learn the best mapping from inputs to labels, and (2) unsupervised learning, where there are no labels and the goal is to discover patterns that reveal unknown but supposedly existing categories within the data.

In recent years, a particularly powerful branch of ML, known as deep learning (DL), has emerged [8], which employs multi-layered neural networks to automatically extract increasingly complex features from raw data, achieving state-of-the-art results in domains such as image recognition, natural language processing, and speech analysis. The DL is widely used in human medicine, where it improves diagnosis, treatment planning, and disease monitoring with measurable benefits for outcomes and costs [9], but its adoption in veterinary medicine is still at an early stage [10]. Most existing review papers focus on AI applications in diagnostics, disease detection, and treatment support [11,12,13,14,15,16,17,18]. A smaller subset of reviews extends this focus to behavioural analysis [2,19,20], while only isolated reviews address animal welfare [21] and, to the authors’ knowledge, there are no review papers that include the prediction of personality traits in companion animals. Yet AI-based solutions carry substantial potential for improving welfare, strengthening owner–animal relationships, and enabling the early identification of behavioural problems.

To address this gap, the present paper provides the first systematic review, conducted using PRISMA methodology, that encompasses not only established AI applications in veterinary diagnostics and everyday care but also the more recent developments in predicting animal behaviour and personality traits. Moreover, our review extends beyond dogs and cats to include exotic companion animals, a group that has received limited attention in existing review literature.

Accordingly, the primary research question of this review is: how are AI methods currently applied in companion animal care, and what are their main limitations, gaps, and future research directions.

The review is divided into 9 sections. Section 2 describes the PRISMA methodology used for the study’s selection of related research work. Section 3 provides an overview of AI applications in veterinary diagnostics and clinical care. Section 4 examines AI applications in everyday companion animal care. Section 5 reviews AI approaches for predicting animal personality, while Section 6 focuses on AI for behavioural trait prediction. Section 7 reviews AI applications in exotic companion animals, and Section 8 discusses several aspects covered in the review and its limitations. Lastly, Section 9 provides conclusions and future perspectives.

## 2. Methodology

The literature search was conducted in accordance with the PRISMA methodology using the Scopus and PubMed databases. These databases were selected as primary sources because of their broad, complementary coverage of biomedical, veterinary, and technical literature relevant to AI applications in companion animals. Additional databases (e.g., Web of Science, IEEE Xplore) were considered but not included due to substantial overlap with Scopus and limited additional relevance for the scope of this review. The completed PRISMA checklist is provided as Supplementary Materials.

Data extraction was performed independently by two reviewers using a predefined data extraction form. Risk of bias and certainty of evidence were not formally assessed due to the heterogeneity of included studies.

The search covered the period from 2020 to 2025 and was performed between January and February 2026. The strategy combined free-text terms and, in PubMed, controlled vocabulary (MeSH terms), using Boolean operators (AND, OR, NOT). Searches were restricted to title, abstract, and keyword fields in Scopus and to Title/Abstract fields in PubMed to improve relevance. Keywords were grouped into conceptual categories, with terms within categories connected by “OR” operators and categories combined using “AND” operators. Consequently, articles were included if they matched at least one term from each category, rather than all listed keywords.

The Scopus search string was:

(“artificial intelligence” OR “machine learning” OR “deep learning” OR “neural network*” OR “AI”) AND (“veterinary medicine” OR veterinary OR “companion animal*” OR pet* OR dog OR dogs OR cat OR cats OR “exotic pet*” OR “small animal*”) AND (behavior OR behaviour OR “animal behavior” OR personality OR temperament OR diagnosis OR “behavioral assessment”).

The PubMed search strategy combined Title/Abstract terms with controlled vocabulary (MeSH terms) to increase sensitivity and specificity of the search, while excluding non-relevant species. The search string was:

(((“artificial intelligence”[Title/Abstract] OR “machine learning”[Title/Abstract] OR “deep learning”[Title/Abstract] OR “neural network*”[Title/Abstract] OR “AI”[Title/Abstract] OR “Artificial Intelligence”[MeSH Terms])) AND ((“veterinary medicine”[Title/Abstract] OR “veterinary”[Title/Abstract] OR “companion animals”[Title/Abstract] OR pets[Title/Abstract] OR dog[Title/Abstract] OR dogs[Title/Abstract] OR cat[Title/Abstract] OR cats[Title/Abstract] OR exotic pets[Title/Abstract])) AND ((behavior[Title/Abstract] OR behaviour[Title/Abstract] OR “animal behavior”[Title/Abstract] OR personality[Title/Abstract] OR temperament[Title/Abstract] OR diagnosis[Title/Abstract]))) NOT ((humans[MeSH Terms]) OR (cattle OR pigs OR swine OR sheep OR goats OR livestock OR poultry)).

All retrieved records were imported into Zotero, and duplicates were removed using the built-in “Duplicate Items” function before screening.

The search was restricted to peer-reviewed journal articles and review papers published in English. The selected time frame (2020–2025) was chosen to capture recent developments in AI technologies, including deep learning, wearable sensors, and real-time behavioural analysis.

Selected grey literature (e.g., official company and organizational websites) was included to complement the scientific literature and provide contextual insights. Only sources containing verifiable and directly relevant information were considered.

**Figure 1:**
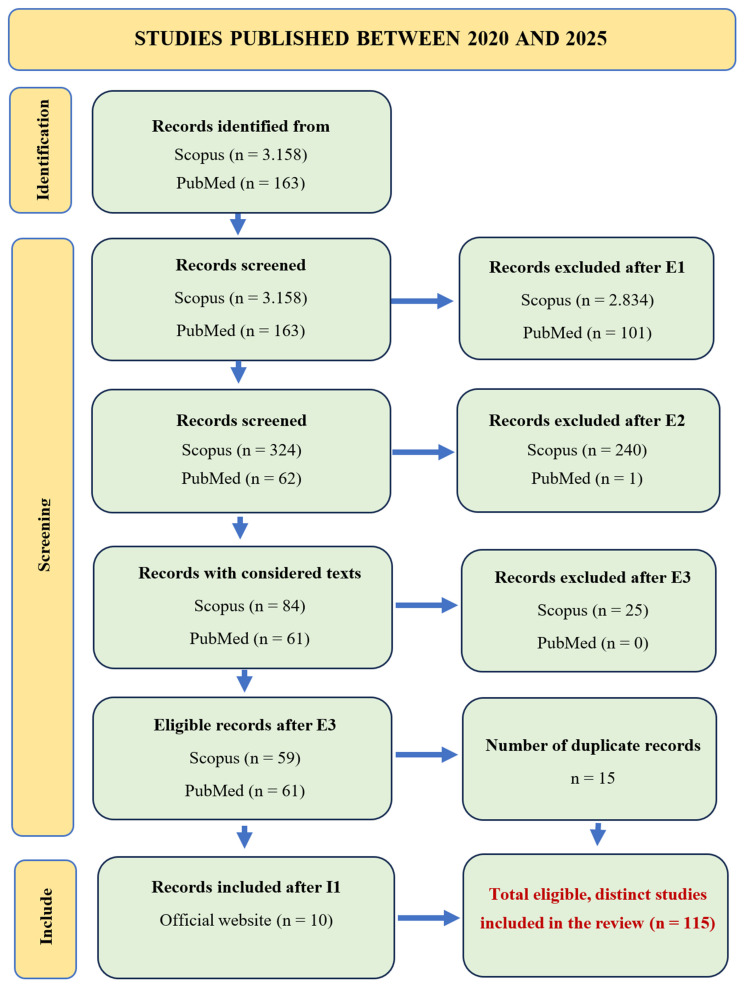
PRISMA Flow Diagram showing the identification, screening, eligibility assessment, and inclusion of studies published from 2020 to 2025.

A total of 3.321 records were identified, with inclusion and exclusion criteria detailed in Table 1 and the selection process illustrated in Figure 1 (PRISMA flow diagram). Following screening, 115 studies were included in the final analysis, of which 10 were review articles. The 115 studies also include 10 references from official websites, which were incorporated narratively to provide a broader theoretical context. The total number of references cited in this review (*n* = 149) includes both studies selected through the systematic screening process and additional sources that were incorporated narratively to enrich the contextual and theoretical framework.

## 3. AI-Driven Advances in Diagnostic and Clinical Care for Companion Animals

Animals cannot communicate discomfort verbally, which makes the early detection of health problems a persistent challenge in veterinary medicine. The integration of AI into veterinary medicine is beginning to transform companion animal healthcare, particularly through ML systems that analyze complex health datasets to improve diagnostic accuracy and support therapeutic decision-making [2,22].

Survey data indicate that veterinarians are generally familiar with AI and optimistic about its integration into clinical practice, particularly younger ones. Also, those already using AI tools report benefits such as increased efficiency, reduced administrative workload, and improved diagnostic processes [23].

In recent years, innovative AI solutions have emerged to support veterinarians across various areas of clinical practice, with many platforms tailored to assist at every stage of the clinical process [24,25,26]. LAIKA, for example, is a system trained on thousands of real clinical cases and designed specifically for veterinary medicine. It can interpret clinical histories, analyse laboratory results, and suggest diagnostic pathways in real time, while being continuously refined by practicing veterinarians [25].

Beyond general practice, AI is also advancing specific fields of veterinary medicine, such as oncology and radiology [27,28]. For example, FidoCure^®^ is an AI-supported precision oncology platform that integrates genomic sequencing with clinical decision-making [27]. A recent study using real-world genomic data from dogs diagnosed with splenic hemangiosarcoma demonstrated that specific mutations were associated with differential responses to targeted therapies. These findings illustrate the potential of AI-driven genomic profiling to guide personalised treatment plans in veterinary oncology and to achieve improved outcomes compared with standard chemotherapy alone [29].

In 2022, the global animal therapeutics and diagnostics market was valued at approximately 58 billion U.S. dollars and is projected to exceed 80 billion U.S. dollars by 2028 [30]. These projections suggest a strong and expanding demand for diagnostic solutions, including AI-based tools. Yet critical questions remain regarding external validation, data quality (e.g., data biases and missing values), ethical considerations (surveillance risks), and the cost-effectiveness of such tools.

**Figure 2:**
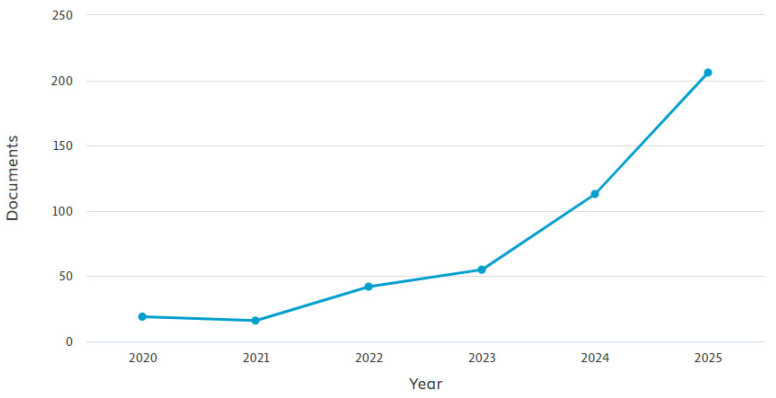
Number of documents retrieved from Scopus using the keywords “veterinary medicine” and “artificial intelligence” by publication year (2020–2025).

Also, scientific interest in the application of AI in veterinary medicine has been steadily increasing. As shown in Figure 2, Scopus-indexed publications combining the keywords “veterinary medicine” and “artificial intelligence” have increased since 2020. In 2020 and 2021, the number of studies was low. This trend might have been influenced by the COVID-19 pandemic, which redirected global research efforts toward urgent public health issues. From 2022 onwards, a steady increase is observed, reflecting the gradual adoption of AI methods in veterinary medicine. In 2024, the number of publications more than doubled compared to the previous year, which demonstrates that scientific interest in such topics has been strengthening year by year, most likely as a result of the increasing implementation of AI solutions across various spheres of life, including veterinary medicine. It is important to note that these data are limited to Scopus and rely on specific keywords, which may overlook relevant studies using alternative terminology; however, they still reflect a broader trend.

AI algorithms can rapidly process complex biomedical data, uncover hidden patterns, and support decision-making, enabling earlier diagnoses, targeted treatments, and more efficient healthcare management. An overview of recent studies (from 2020 to 2025) that apply to AI-driven advances in diagnostic and clinical care for companion animals is presented in Table 2.

As shown in Table 2, AI has clearly transformed veterinary medicine by reshaping diagnostic and therapeutic approaches, advancing personalized patient care, and driving the development of numerous AI-based solutions. Importantly, both the quantity and quality of research are evidently keeping pace with this development. However, many investigations are still in their early stages. Also, most scientific studies on AI applications in veterinary medicine come from radiology. This is likely because there is an abundance of suitable medical images and data for creating and validating AI models, and because the intrinsic properties of radiological images align well with well-established computer vision methods.

Although AI in veterinary medicine offers promising solutions, several barriers and challenges remain. The ethical use of AI in veterinary medicine raises issues of data ownership, confidentiality, and security, while the absence of a regulatory framework, like in human medicine, underscores the need for clear regulations to ensure responsible implementation [4]. A further challenge concerns data accessibility, as many veterinary clinics function independently and lack the infrastructure for centralized data sharing [71]. For training a high-performing algorithm, a large amount of data is required. Another significant concern is the uneven geographical distribution of scientific studies, which poses a challenge for the generalization of the results. When research is concentrated in specific regions, AI-driven veterinary solutions risk being developed and validated on datasets that may not adequately reflect diverse clinical settings worldwide. Moreover, the issues of AI model explainability and the choice of interpretable AI methods plague this field just as it does with human medicine [72,73]. Most models do not lend themselves to easy explainability, which prohibits their general acceptance among veterinarians [65]. Survey data showed that veterinarians also have concerns about reliability, accuracy, data security, costs, and insufficient training for using AI in veterinary medicine [23].

Therefore, the integration of AI-based systems into routine veterinary practice requires further studies and evaluation in terms of data standardization and generalization, interoperability, and alignment with existing clinical decision-making processes. Another important aspect of the successful and responsible integration of AI into veterinary medicine is the education of veterinarians on AI-based solutions that enhance veterinary medicine by improving diagnostic accuracy, optimizing therapeutic strategies, and supporting personalized patient care.

## 4. AI Tools Supporting Everyday Care and Early Health Monitoring in Companion Animals by Owners

In households, AI-based systems can contribute to early health monitoring by continuously tracking behavioral and physiological parameters such as food intake, activity levels, and movement patterns. Deviations from baseline patterns can be automatically detected, allowing early identification of potential health issues such as reduced appetite, lethargy, or mobility impairments, often before clinical signs become evident to the owner. However, the effective adoption of these technologies is strongly influenced by user attitudes and trust in AI.

People do not share the same attitudes toward AI, and these views vary from country to country. The most positive perception toward AI is in China, where 80% of respondents agree with the statement, “Products and services using AI make me excited.” In contrast, only 34% of respondents in the U.S. agree with this statement, while 64% say, “Products and services using AI make me nervous.” [30]. Despite this, AI is transforming everyday life, including the ownership of animals. Therefore, AI-based solutions are emerging as tools that support responsible ownership and care of companion animals, and many software are designed as monitoring solutions for owners [74,75,76].

In addition to health monitoring, recent trends highlight the growing use of AI in companion animal nutrition and feeding management, including personalized diet recommendations, smart feeding systems, and behaviour monitoring during eating, which are essential components of everyday animal care [19].

Unlike Table 2, which presents studies on AI models that support veterinarians in clinical diagnosis and treatment planning, Table 3 provides an overview of recent studies (2020–2025) on the application of AI in everyday care and early health monitoring by owners of companion animals. Their focus is on monitoring, prevention, and early symptom recognition, not clinical diagnosis.

Although AI solutions can contribute to responsible ownership, they also pose significant challenges to ensuring the welfare of companion animals. The risks include misinterpretation of health data, overreliance on automated recommendations, and delayed veterinary consultation [3]. Many owners still lack adequate knowledge about the species-specific needs of their animals, and unrealistic expectations further compromise animal welfare [82]. To reduce the risks associated with using such applications in companion animal care, owners should undergo training that will provide not only guidance on the responsible use of AI but also basic knowledge about companion animal keeping, care, health, and species-specific needs. In this way, the owner–animal bond can be strengthened, and the animal’s welfare and the owner’s well-being enhanced. According to the annual PDSA Animal Wellbeing Report from 2022 [83], only 14% of owners had heard of the 5 Welfare Needs before completing the survey. The same study revealed that around 28% of owners reported using the internet when choosing a pet, 20% did no research at all and only 6% sought advice from a veterinary professional. This highlights a substantial gap in informed decision-making before acquiring a companion animal.

The importance of education about AI is clear in the fact that 50% of people feel nervous when it comes to its use [30]. Since caring for companion animals can already be a source of stress for their owners, misunderstanding and mistrust of AI tools could make that stress even worse. Also, globally, fewer than half of people (47%) trust that companies that use AI will protect their personal data [30]. This highlights not only the need to educate the public about this issue, but also the responsibility to invest in stronger protection of personal information. Without such efforts, distrust in AI tools will persist and may even intensify. In addition, veterinarians, as trusted experts, may play a crucial role in reassuring clients about the use and benefits of AI-based tools, provided that they are adequately educated and trained.

It is essential to note that attitudes toward AI vary globally [30,84], suggesting that the acceptance of AI-based tools in animal ownership likely depends on cultural perceptions and trust. While some owners may readily adopt AI solutions for monitoring and enhancing the health of their animals, others may remain cautious, underscoring the importance of transparency, education, and evidence-based validation in promoting the responsible use of AI solutions in everyday pet care. Therefore, it is necessary to examine the level of understanding and knowledge about AI tools across different countries. Without identifying these differences, educational initiatives and policies risk being ineffective or even counterproductive.

In the human–animal relationship, it is important to consider not only human well-being but also the welfare of the animal. Focusing on just one side of this relationship risks creating an unbalanced and ethically problematic approach. Therefore, it is important to note that animals may be particularly vulnerable to harm from AI, given their weaker social, moral, and legal protections compared to humans [85]. Therefore, its design and use must be shaped through ongoing dialogue among veterinarians, developers, and owners. Therefore, interdisciplinary studies involving veterinarians, ethicists, policymakers, technologists, and owners are crucial to ensure that AI integration improves the everyday care for companion animals.

## 5. AI in Predicting Animal Personality

In psychology, personality reflects consistent patterns of behaviour, emotion, motivation, and thought. It strongly shapes human life choices, health, well-being, and individual preferences, making the automatic detection of personality traits highly valuable for practical applications [86]. In human psychology, AI has already been successfully applied to predict personality traits [87,88,89]. Established frameworks such as the Myers–Briggs Type Indicator (MBTI) and the Big Five Inventory (BFI) remain among the most widely used and validated approaches for assessing personality, classifying individuals according to consistent patterns of thought, emotion, and behaviour [90]. The use of AI for predicting animal personality is a relatively new research direction, with only a small number of studies published to date.

Personality similarity between companion animals and their owners may strengthen the human–animal bond, enhancing both animal welfare and human well-being [91,92]. Studies indicate that companion animal personality plays an important role in the process of owner selection. King et al. [93] found that the “ideal dog” for Australian owners was described as medium-sized, short-haired, neutered, safe with children, fully housetrained, friendly, obedient, and healthy. Desired behaviours included coming when called, staying within the property, enjoying physical affection, and showing attachment to the owner. But, in addition to the importance of companion animal personality for owners, understanding individual differences in animal personality enables more tailored care for the animal. For example, active cats require greater physical and mental stimulation, whereas timid individuals function better in calm and predictable environments [94]. Therefore, increasing efforts are being directed toward developing new approaches for recognizing the personality of animals. Recent studies are focusing on the role of AI in this context, as presented in Table 4.

Importantly, these approaches have clear practical value. In assistance dog programs, many dogs are identified as unsuitable only after extensive and costly training. AI-based predictive models using behavioral assessments (e.g., C-BARQ and standardized temperament tests) can identify low-probability candidates early, with accuracies of up to 85–92%. This enables earlier decision-making, reduces training costs, and improves overall program efficiency [95]. This suggests that AI can help improve training outcomes by identifying suitable candidates earlier and making the training process more efficient.

**Table 4:** Overview of recent studies (2020–2025) examining the application of AI in predicting animal personality.

Type of Data	Dataset	AI Model	Species	Conclusion	Reference
Tabular	Behavioral trait scores	ML, Logistic Regression, Support Vector Machine, Random Forest	Dogs	While supervised models showed good performance in identifying dogs that successfully entered training, their ability to distinguish those that were eliminated was limited. Feature selection methods identified key traits, including olfaction, possession, confidence, and initiative, as important predictors of success. These findings highlight the importance of specific tests, environments, behavioural traits, and developmental timing in detection dog selection, and demonstrate the potential of AI approaches to guide future research on cognitive, emotional, and environmental factors.	[96]
Tabular	C-BARQ data	ML, K-means clustering, decision tree	Dogs	This study applied ML to predict canine personality. K-Means clustering revealed five personality types, and decision trees achieved 99% accuracy. These methods show promise for improving dog selection and training, though further validation is needed.	[97]
Tabular	C-BARQ data and data from two assistance dog training organizations	ML, DL, several classifiers	Dogs	These findings highlight the importance of model choice and dataset structure, with traditional ML proving most suitable for early, practical decision-making in assistance dog training.	[98]
Tabular	C-BARQ data and OCEAN or Big Five Test data	ML, K-means clustering, XGBoost classifier	Dogs	This study aims to improve adoptions by matching dogs and humans based on personality traits. By clustering over 12,000 dogs into personality types and classifying human profiles, the approach identified optimal pairings, highlighting mental compatibility as a key factor in successful relationships and well-being.	[99]
Tabular	C-BARQ and MCPQ-R data	ML, multivariate logistic regression	Dogs	The ML models based on C-BARQ and MCPQ-R data showed similar predictive performance (AUC 0.84–0.85), with MCPQ-R proving a reliable alternative for early prediction of assistance dog suitability.	[100]
Tabular	Assistance Dog Test Battery ethogram data	ML, multivariate logistic regression	Dogs	The ML models based on behavioral test battery data successfully predicted assistance dog training outcomes, supporting their use for early selection and cost reduction in training programs.	[101]
Time series	Patchkeeper device (Nokia Bell Labs, Murray Hill, New Jersey, USA)	ML, several classifiers	Dogs	This study trained ten machine-learning models using activity data from wearable sensors to predict dog personality, demonstrating the potential of wearables for assessing pets’ psychological traits.	[102]

Although cats are one of the most popular companion animals, the value of personality assessment in the management and care of cats has received relatively little attention in research [103]. Despite AI’s demonstrated applicability in dog personality research, no study has yet explored its use for assessing personality in cats under everyday household conditions. However, as we mentioned, several studies focus on using a large amount of owner-reported data, such as C-BARQ, for training AI-based models for predicting behaviour traits and personality in dogs. Therefore, using similar data on cats may serve as a good basis for future research. Mikkola et al. [94] analyzed data from over 4300 cats using owner-completed questionnaires and identified seven stable factors, five related to personality and two to behavioral problems, confirming the usefulness of owner assessments in studying individual differences. While questionnaires carry a risk of subjective bias, their reliability and validity can be significantly improved through clear instructions, standardized contexts, and large sample analysis [104]. The Feline Behavioural Assessment and Research Questionnaire (Fe-BARQ), developed by James A. Serpell, provides a standardized method for assessing feline temperament and behaviour based on owner reports [105]. It has proven to be a comprehensive, consistent, and valid tool for evaluating both personality and behavioral disorders in domestic cats [106]. The Fe-BARQ measures 23 key behavioral traits, including activity, sociability, vocalization, attention-seeking, aggression, fear responses, separation-related behaviour, trainability, predatory tendencies, grooming habits, and elimination preferences. In addition, it records 14 miscellaneous behaviours of interest to cat owners, offering a broad overview of feline temperament [105]. The Fe-BARQ dataset has been collected globally, which makes it more suitable for data training, containing less bias when compared to questionnaires collected on a regional level. Therefore, the use of the Fe-BARQ data could yield promising results in predicting cats’ behaviour and personality traits with the support of ML.

## 6. AI in Predicting Companion Animals’ Behaviour Traits

Animal welfare is a complex, multifactorial concept shaped by diverse scientific, ethical, social, and cultural influences. As human impact is central, meeting the welfare needs of companion animals throughout their lives is widely recognized as a key human responsibility [107]. Insufficient owner awareness of species-specific animal needs, as well as unrealistic expectations, are among the main causes of compromised welfare [82]. Chronic stress in companion animals can lead to physiological imbalances, behavioral issues, and compromised welfare [108,109]. These conditions are often difficult to reverse and may result in the relinquishment of animals to shelters or their abandonment [82]. Today, there are over 100 million abandoned companion animals just across Europe [110]. Also, behavioural problems in companion animals may lead to poorer owners’ well-being [111]. Therefore, early recognition of such problems is of great importance not only for physical health and welfare but also for the well-being of owners. Also, in dogs, individual differences in cognitive and behavioural traits are well documented, highlighting the importance of understanding and characterizing these traits for various applications [112]. Therefore, research on the application of AI to the companion animal behaviour field is also advancing. An overview of recent studies (from 2020 to 2025) that apply AI in predicting companion animals’ behaviour traits is presented in Table 5.

**Figure 3:**
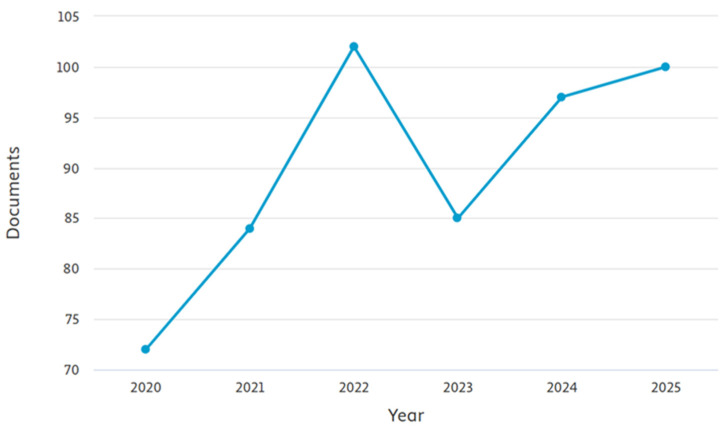
Number of documents retrieved from Scopus using the keywords “cat”, “feline” and “behaviour” by publication year (2020–2025).

**Figure 4:**
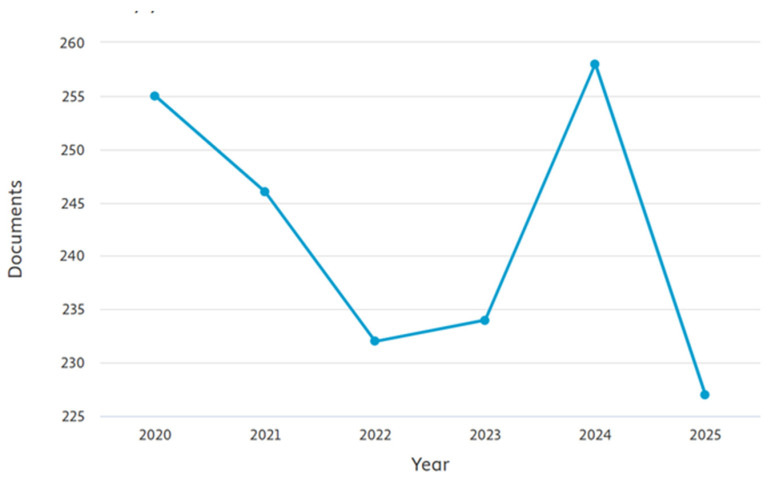
Number of documents retrieved from Scopus using the keywords “dog”, “canine” and “behaviour,” by publication year (2020–2025).

It is important to note that, except for one study on hamsters, the available literature is almost entirely restricted to dogs and, to a lesser extent, cats. Similar to the use of AI in personality assessment, studies on the role of AI in monitoring the behaviorof cats are also underrepresented compared to those focusing on dogs, because the scarcity of accessible datasets and specialized hardware is even more significant [120]. However, despite the prevalence of behavioral issues in cats, their incidence and the genetic and environmental factors contributing to them remain underexplored. Figure 3 and Figure 4 highlight a clear imbalance in research output, with studies on dog behaviour (≈approximately 230–260 papers/year) consistently outnumbering those on cats (≈approximately 70–100 papers/year). While cat research shows gradual growth, it remains underrepresented compared to the sustained focus on dogs, underscoring the need for greater attention to cat behaviour studies. Also, the most available data on cats’ behaviour come from studies on free-roaming cats, colonies, or shelter populations, whereas household cats are underrepresented in the literature [106,126].

Although behaviour recognition studies focus more on dogs than cats and more open-source datasets are available for dogs, the overall amount of research on cats’ behaviour remains limited [120]. The first essential step is the systematic collection of large datasets combined with a thorough understanding of the species-specific needs of household cats. Without this foundation, any integration of predictive models remains limited, as robust behavioural prediction requires both sufficient data volume and biologically meaningful context.

## 7. AI in Exotic Companion Animal Care

As observed by Ostović et al. [127], although exotic pets are often defined as non-native or non-domesticated species, in veterinary practice, the term generally refers to any companion animal other than a dog or a cat. These pets are increasingly traded worldwide, now including over 500 bird and reptile species [128]. While most people, and veterinarians in particular, are well acquainted with the needs and disorders of domesticated animals, much less is known about exotic species, whose requirements are often far more specific [129,130]. For example, Azevedo et al. [131] reported that 85% of Portuguese reptile owners failed to meet at least one basic husbandry requirement and frequently misinterpreted stress behaviours as normal. Also, amphibians, despite their popularity as companion animals, are likewise undervalued in terms of emotional capacity, underscoring the need for further research on their sentience and welfare [132].

Today, an increasing number of studies are focusing on the application of AI in biology, physiology, and diseases of free-living birds, reptiles, and amphibians [133,134,135]. This research primarily targets free-living taxa, but such technological advances could be applied to captive environments, such as zoos, laboratories, and exotic pet care. In parallel, commercial AI applications for companion animal care, such as the AI assistant Daisy, are emerging as integrated platforms that provide real-time, personalized guidance across different stages of pet ownership, including feeding, behaviour, and health monitoring. Although currently limited to dogs and cats, such systems illustrate a broader trend toward scalable, user-oriented AI solutions that could be extended to exotic species in the future, where knowledge gaps among owners are even more pronounced [136]).

Despite growing interest, empirical studies on AI in captive exotic species remain extremely scarce. A study by Parra et al. [137] demonstrated that AI-based image analysis can reliably detect parasitic infections in captive reptiles, achieving over 94% accuracy in distinguishing several common parasitic agents from microscope stool images. These findings highlight the potential of convolutional neural networks to support more efficient and accurate diagnosis in exotic pet medicine. Also, in reptiles, AI has been applied to automate the acquisition of body temperature, a key parameter in laboratory and clinical settings [138]. In this work, computer vision was used to identify lizard body parts in RGB footage and match them to corresponding thermal data, enabling automated temperature recording without repeated manual handling—the method not only improved accuracy and consistency but also reduced animal stress compared to traditional techniques.

It has been recognised that exotic pets can present health and safety risks and pose threats to the environment and biodiversity [139]. A study by Liu et al. [128] proposed a novel method for recognizing fine-grained images of small exotic pet snakes in complex backgrounds, based on an improved SimCLR framework. The relevance of this research lies in the fact that the exotic pet trade is a major driver of alien species invasions, and improper introductions or inadequate management can lead to severe ecological consequences. In a study conducted on the Canary Islands, where invasive reptile species pose a growing threat to local ecosystems, which include several endemic reptiles, researchers tested DL models for automatic reptile identification. The best-performing model, based on EfficientNetV2B3, achieved a mean accuracy of 98.75%, demonstrating the potential of AI for supporting early detection and management of invasive reptile populations [140].

Beyond diagnostic applications, AI can also be applied for behavioral monitoring, as changes in behaviour are often among the earliest indicators of health problems and compromised welfare. For instance, Shibanoki et al. [115] developed a DL system to monitor behavioral and internal state changes in a pet hamster. The system successfully identified deviations from normal daily activity. It generated alerts in response to unusual stimuli, demonstrating the potential of non-invasive, video-based monitoring approaches for the early detection of stress and health-related alterations.

The reasonable and ethical development of AI models and their application in exotic companion animals may significantly contribute to improving their welfare, as well as environmental protection. To develop such models for monitoring the microclimate conditions, health and behaviour of exotic animals in home environments requires extensive, high-quality datasets from owners, including images, videos, and structured questionnaires. Gathering such information through standardized tools and digital platforms would provide a robust basis for advancing AI solutions in this field. Additionally, to improve the welfare of exotic companion animals, the crucial step is educating owners on the basic, species-specific needs of their animals. Without a basic understanding, overreliance on AI solutions can lead to the misinterpretation of health data and delayed veterinary consultations.

## 8. Discussion

In recent years, there has been a rapid growth in interest in AI research across various fields. However, this increasing attention has also been accompanied by heterogeneity in the use of AI-related terminology. Interestingly, although the search strategy explicitly required AI-related keywords, 25 studies did not use any AI methods, including neural networks, ML, or DL. This discrepancy suggests that AI-related terms are sometimes used in a broader or conceptual sense of any automation or statistical reasoning method rather than to describe the application of a more specific AI method.

Despite rapid technological progress, we have shown that current AI applications in veterinary care remain substantially limited by the nature and quality of available data. Most studies rely on small datasets, sometimes acquired for a specific purpose, which restricts statistical power and increases the likelihood of overfitting. These constraints mean that AI models built from such datasets often learn narrow patterns tied to specific individuals, breeds, environments, or recording conditions rather than species-wide behavioural or physiological features. In addition, veterinary medicine should address the challenge of multi-species AI model generalisation for everyday care. Namely, anatomical, behavioural, and vocal differences across species (and even across breeds within species) make it difficult for a single model to perform reliably for several species in a clinical or home environment. Although transfer learning AI approaches hold promise for mitigating this problem, their development in veterinary science is still in an early stage and is not yet sufficient to bridge major interspecific gaps in realistic settings. Another major limitation is the lack of external validation, as we have shown that most published studies evaluate models on a single dataset or within a single clinical setting. Only in rare cases does model validation exist on external datasets. Without testing independent populations, clinics, imaging systems, environments, or owner-collected data, the robustness of these models remains uncertain, and performance metrics reported in the literature will likely overestimate real-world applicability. From a practical perspective, it is helpful to recognise that not all AI solutions described in the literature are at the same stage of development. Most of the identified studies present experimental models that were developed and tested within a single research setting, without wider clinical adoption. A smaller number of applications are already available as commercial products, yet independent scientific validation is often limited or not publicly reported.

It should be noted that cross-clinic variability, ranging from differences in diagnostic equipment to imaging quality, can cause substantial performance drops when AI models are deployed outside the environment in which they were trained. As a result, although promising prototypes exist, the current generation of veterinary AI tools remains fragile, and the scarcity of large, diverse, and well-validated datasets limits their clinical or everyday applicability.

These matters are recognized and actively discussed in the veterinary community and more broadly. On the “Tech-Enabled Tomorrow” panel at the 2025 AVMA Veterinary Business and Economic Forum, there was a discussion about the perks and pitfalls of AI in veterinary medicine. As stated, the adoption of AI in veterinary medicine requires comprehensive regulatory frameworks encompassing algorithm transparency and explainability, robust external validation, sound data governance, and clearly defined professional responsibility and liability. These measures are essential for ensuring patient safety, maintaining the trust of animal owners, and encouraging responsible use of AI in clinical practice. In future veterinary clinics, AI is expected to manage administrative and organizational tasks, allowing veterinarians to focus on diagnosis, treatment, and empathetic patient care. However, the absence of standardized education in AI presents a significant challenge. In addition, responsibility for educating pet owners on the appropriate use of AI-based tools remains unclear. While veterinarians may play a key role, it likely also involves developers and service providers. The lack of clearly defined roles may limit safe and effective use and highlight the need for structured guidelines and education. Without adequate AI literacy, there is a risk of overreliance on technology, which can compromise clinical judgment and decision-making [141]. This highlights a critical need to integrate AI literacy into veterinary education to ensure the responsible and informed use of these technologies. The integration of AI into the education of future professionals has been increasingly recognized, as evidenced by the scientific literature. As reported by Reagan et al. [142], AI-based applications in veterinary medicine are becoming more widely available, and veterinary students show strong interest in these technologies and their relevance to future clinical practice. Yet training in this field remains lacking in many veterinary curricula.

Furthermore, the rapid advancement of AI is transforming decision-making across sectors, including healthcare. Although AI systems do not possess legal agency, their increasing technical autonomy in decision-support processes complicates the attribution of legal responsibility, representing a major global challenge [143].

As we have shown, emerging AI systems increasingly integrate wearable devices into companion animal care, enabling continuous monitoring of activity, mobility, and physiological proxies for disease detection and welfare assessment. However, challenges related to data standardisation, device heterogeneity, and limited validation across species constrain their broader clinical applicability. Particular caution is required in cats, as even “safety collars” do not fully prevent severe incidents [144], highlighting the need for alternative monitoring approaches. Importantly, these limitations also reflect a broader gap, the insufficient representation of feline behaviour in AI research. Despite their prevalence, cats remain underrepresented in behavioural datasets, particularly in household environments [106,126]. Their behaviour is often subtle, context-dependent, and shaped by genetic, personality, and early-life factors [94,145,146], while indoor living conditions may further contribute to stress and behavioural disorders [108,109,147].

Nutrition is increasingly recognized as a key determinant of companion animal health, longevity, and overall quality of life [148]. At the same time, growing demands for safe, high-quality, and traceable diets, together with the increasing complexity of nutritional management, underscore the need for advanced, data-driven approaches. In this context, AI has emerged as a promising tool for optimizing feeding strategies and enabling more precise and personalized nutrition. Its potential is further supported by recent applications in food safety, where sensor-based technologies and ML models have demonstrated high accuracy in detecting contaminants such as mycotoxins in pet food [149]. Future research is therefore needed, particularly from a practical perspective, as it could provide pet owners with reliable, evidence-based tools to optimize nutrition and improve long-term health outcomes.

Moreover, across healthcare, including veterinary medicine, much of the available data remains unstructured and requires manual processing for research purposes, which limits efficiency and scalability [148]. Natural language processing (NLP) offers a scalable solution by enabling the automated extraction of relevant information from large volumes of textual data. However, the clinical adoption of NLP is hindered by limited access to suitable datasets and the need for specialised domain expertise [149]. Addressing these challenges requires improved data availability, greater standardisation of clinical text, and the active involvement of veterinary domain experts in model development. Moreover, as current NLP models, including large language models, are not specifically trained on veterinary clinical texts and health records, future research should prioritise the development of domain-specific resources and externally validated models trained on diverse veterinary datasets to ensure robust and clinically relevant NLP applications in veterinary practice.

**Figure 5:**
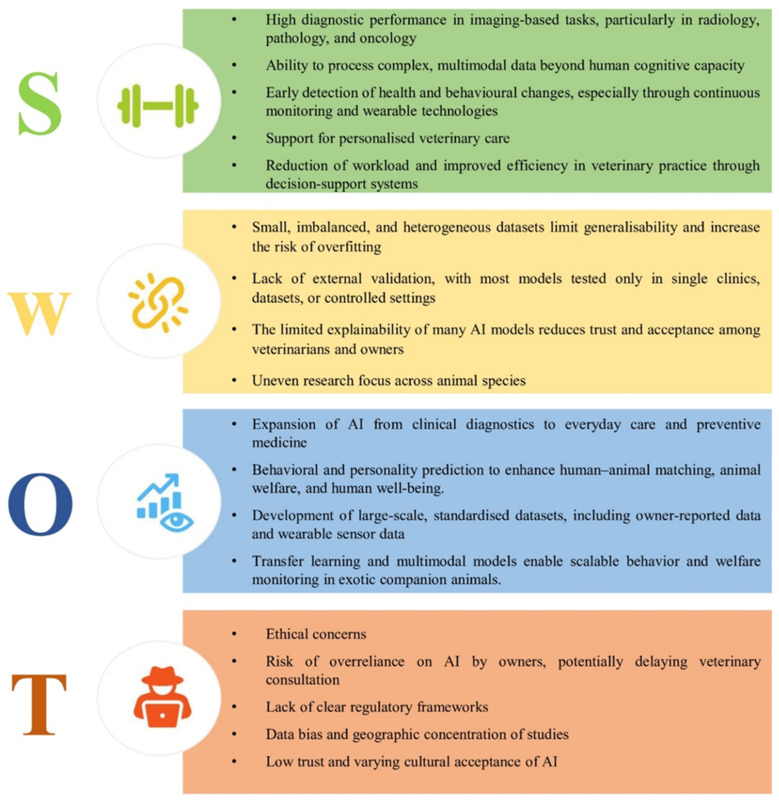
Strengths, Weaknesses, Opportunities, and Threats (SWOT framework) of AI in Companion Animal Care.

To synthesise the key points emerging from the reviewed literature and to contextualise the potential and limitations of AI applications in companion animal care, a SWOT (Strengths, Weaknesses, Opportunities, and Threats) framework is used as an interpretative tool and is depicted in Figure 5. Taken together, this SWOT analysis shows a clear disconnection between the strong technical potential of AI in veterinary medicine and its current readiness for broad clinical and everyday-care adoption. Namely, while AI systems demonstrate robust performance in data-intensive diagnostic domains, their use in applications supporting daily animal care, behaviour monitoring, and welfare assessment remains somewhat limited by rather small and biased datasets, insufficient external validation, and uneven research attention across species. The limitations are particularly important for companion animal welfare, where variability in environments, owner behaviour, and species-specific traits is high and thus requires elaborate AI solutions. Moreover, persistent challenges related to explainability, ethical considerations, and the absence of clear regulatory frameworks continue to impede implementation beyond controlled clinical settings.

## 9. Conclusions

In conclusion, AI holds significant promise for advancing companion animal care, from diagnostics to behavioural and personality assessment. Nevertheless, its broader implementation is constrained by limited and heterogeneous datasets, insufficient external validation, species imbalance, and unresolved ethical and regulatory challenges. Future efforts should focus on developing large-scale, standardised datasets, ensuring robust cross-context validation, and promoting interdisciplinary collaboration. Strengthening model interpretability and AI literacy among veterinary professionals will be essential to enable responsible, evidence-based integration that ultimately enhances animal welfare and clinical decision-making.

## Figures and Tables

**Table 1:** Exclusion and inclusion criteria for the review.

No.	Exclusion (E) and Inclusion (I) Criteria
E1	Papers unrelated to veterinary medicine, companion animals and AI (e.g., human medicine, engineering, biochemistry, mathematics, physics and astronomy, material science, decision science…).
E2	Papers not involving companion animals (dogs, cats, exotic pets) or studies focused exclusively on animal models or farm animals (e.g., livestock, horse, cattle, sheep, pig).
E3	Papers not applying AI methods (e.g., studies without machine learning, deep learning or neural networks…).
I1	Relevant grey literature (company websites).

**Table 2:** Overview of recent studies (from 2020 to 2025) focusing on AI-driven advances in diagnostic and clinical care for companion animals.

Field	Type of Data	AI Model	Species	Sample Size	Conclusion	Reference
Cardiology	Time series	ML (supervised learning)	Dogs	73	The ML combined with Poincaré plot analysis demonstrated accurate classification of sinus node dysfunction, highlighting its potential for improving diagnostic precision in veterinary cardiology.	[31]
Clinical decision support	Tabular	ML (multi-model platform)	Dogs	Unspecified	The Anna platform enables real-time integration of ML models with EHR systems, supporting diagnostic decision-making and facilitating broader clinical adoption of AI tools.	[32]
Dermatology	Images	DL, YOLOv5 detection network	Dogs	Unspecified, 626 images in total	This AI-based detection model for identifying healthy ear canals, otitis, or masses in the canine ear canal has the potential for application in the field of veterinary dermatology, but an external validation study is needed before clinical deployment.	[33]
Dermatology	Time series	ML, several classifiers	Dogs	72 VOCs sampled from breath and hair samples	The proposed platform integrates volatile organic compound (VOC) sensing, ML–based approaches, and cloud-native infrastructure for the non-invasive diagnosis of leishmaniasis in dogs, demonstrating clinical utility, owner acceptability, research value, and scalability for broader applications in veterinary diagnostics.	[34]
Diagnostic	Images	DL, DenseNet201	Dogs/bulldogs	1020 nostril images, 190 real, others synthetic	The model achieved human-comparable diagnostic performance in the classification of bulldog stenosis degree, thereby supporting improved treatment planning and promoting animal welfare.	[35]
Diagnostic	Images	Deep-learning, convolutional neural network algorithms	Dogs and cats	Up to 92 dogs’ and 69 cats’ CBC images, depending on the trial	This DL method achieved performance comparable to clinical pathologists and complemented automated CBC analysis by confirming cell counts, detecting platelet clumps, and assessing polychromatophils count.	[36]
Diagnostic	Tabular	ML, AdaBoost classifier	Dogs	1025	The ML model accurately screened dogs for hypoadrenocorticism using routine clinicopathologic data, demonstrating high predictive performance with acceptable false-positive rates in a low-prevalence population.	[37]
Diagnostic	Time series	ML, two kNN models and one decision tree model, with majority voting	Dogs	366 audio samples (148 dogs)	The ML models enabled objective classification of brachycephalic obstructive airway syndrome using respiratory audio recordings, achieving good diagnostic performance and supporting a more standardized assessment compared to traditional methods.	[38]
Diagnostic	Tabular	ML, several regressors, best results with AdaBoost regressor for cats and support vector machine for dogs	Dogs and Cats	400	The ML models accurately predicted core body temperature from surface measurements, offering a non-invasive alternative for clinical temperature assessment in companion animals.	[39]
Diagnostic	Tabular	ML, multivariable logistic regression	Dogs	939 (398 cases, 541 controls)	A clinical prediction model based on electronic health records demonstrated good performance in identifying dogs with Cushing’s syndrome, supporting its use as a decision-support tool in veterinary practice.	[40]
Diagnostic	Images	ML, DL, best results with ResNet152 + XGBoost classifier, compared with several ML classifiers	Dogs	210	A DL approach accurately classified estrous cycle stages from vaginoscopic images, supporting its use as a diagnostic tool for reproductive management.	[41]
Diagnostic	Tabular	ML, LASSO regression	Dogs	6287	ML models demonstrated good predictive performance for future Cushing’s syndrome diagnosis, indicating potential for early detection and support in veterinary clinical decision-making.	[42]
Nutrition management	Tabular	ML, decision tree	Cats	101	A decision tree model identified infectious, chronic, or acute disease status, age, and body condition score as key predictors of vitamin B6 deficiency, supporting targeted supplementation strategies.	[43]
Oncology	Tabular	ML, support vector machine	Dogs	45 dogs/69 masses	With further development, this system could serve as a decision-support tool for distinguishing benign lesions from those requiring further diagnostics, while also providing proof-of-concept for prospective cancer diagnosis trials in companion dogs using advanced thermodynamics and ML.	[44]
Oncology	Images	DL, two-stage (U-Net and EfficientNetB5)	Dogs	350	The two-stage tumor classification results demonstrate the feasibility of AI-based methods as supportive tools in diagnostic oncologic pathology, with potential applications across other species and tumor types.	[45]
Oncology	Images	DL, UNet++	Dogs	96	This study highlights the potential of AI–based methods in the morphometry of nuclear pleomorphism in canine cutaneous mast cell tumors. However, further studies are required to validate the existing findings, assess the robustness of different algorithms, and evaluate their applicability across diverse clinical contexts.	[46]
Oncology	Images	DL, nnUNet v2	Dogs	200 CT cases	The algorithm achieved high accuracy, demonstrating the potential of automated CT-based segmentation of hepatic masses in dogs.	[47]
Oncology	Dataset	ML, proprietary classification algorithm	Dogs	1947	An integrated ML test combining cell-free DNA quantification and next-generation sequencing achieved high specificity and moderate sensitivity for multi-cancer detection, supporting its use in early screening	[48]
Pathology	Images	DL, two-stage convolutional neural network (CNN)	Dogs	32	The study demonstrated that variability in manual mitotic counts originates from differences in area selection and proposed computer-based support to enhance agreement.	[49]
Pathology	Image	DL, CNN	Cats	383	The AI model, under pathologist supervision, provides a reproducible and objective whole-slide assessment of feline intestinal lymphocytes, with potential to improve diagnostic accuracy in chronic enteropathy.	[50]
Pathology	Dataset	ML, multivariate logistic regression	Dogs	50	The model demonstrated moderate accuracy in classifying mast cell tumor grades, with potential utility in supporting diagnosis, staging, and clinical management.	[51]
Pathology	Dataset	ML, several clustering methods and classifiers	Dogs	113	Metabolomics-based ML demonstrated excellent performance in differentiating hepatopathies, indicating potential as a diagnostic and prognostic tool.	[52]
Preventive care	Time series, images	ML, associative neural network	Dogs	30	The Health Score, validated against veterinary diagnoses with 87.5% concordance, proved reliable for assessing canine health through daily activity monitoring and may assist owners in evaluating their companion animals’ condition.	[53]
Prognostic	Tabular	ML, multilayer perceptron (MLP) network	Cats	218	A model identified cats aged ≥7 years at risk of developing chronic kidney disease within 12 months, enabling more frequent monitoring than annual checks, based on single-visit clinical variables.	[54]
Prognostic	Tabular	ML, decision tree	Cats	46	Decision tree-based models help predict short- and medium-term survival in cats with acute-on-chronic kidney disease.	[55]
Prognostic	Tabular	ML, random forest	Dogs	165	The ML applied to early-life rectal microbiome data accurately predicted fading puppy syndrome–related death, identifying specific microbial signatures associated with increased mortality risk.	[56]
Radiology	Images	DL, DenseNet121	Dogs and cats	22,000	This method can assist in detecting various lesion types but does not provide a diagnosis. Given its strong overall performance, it may serve as a supportive tool in evaluating primary thoracic lesions for general practitioners while awaiting radiology reports.	[57]
Radiology	Images	DL, CNN	Dogs	792 patients, each evaluated by a thoracic radiograph and a contemporaneous echocardiogram	This work presents proof-of-concept for utilizing DL in veterinary computer-aided diagnosis with application to canine left atrial enlargement.	[58]
Radiology	Images	DL, CNN	Dogs	11,759	The proposed DL models show potential as tools for hip screening protocols, provided that classification performance for hip dysplasia is enhanced through the use of larger datasets and model optimization.	[59]
Radiology	Images	DL, CNN (several models)	Dogs	481	Proprietary AI-based software for screening thoracic radiographs in dogs with suspected cardiogenic pulmonary edema can support short-term clinical decision-making when a radiologist is unavailable.	[60]
Radiology	Images	DL, fine-tuned ResNet-50 CNN	Dogs	6028 latero-lateral and 4053 sagittal radiographs	This AI-based algorithm is a promising tool for improving the accuracy of radiographic interpretation by identifying technical errors in dogs’ thoracic radiographs.	[61]
Radiology	Images	DL, CNN	Dogs	36 canine head and neck patients and 197 humans	The DL–based automatic gross tumor volume segmentation using CNNs trained on dog data alone or via cross-species transfer learning shows promise for future radiotherapy applications in dogs’ head and neck cancer.	[62]
Radiology	Images	DL, CNN, EfficientNet	Dogs	7229	The model demonstrated robust performance in differentiating normal and abnormal canine elbow radiographs, with uncertainty estimation enabling identification of cases requiring human review.	[63]
Radiology	Images	DL, CALCurad algorithm	Dogs	139	The results suggest that the software can predict urolith composition in dogs, supporting clinical decision-making between medical and surgical management and illustrating the utility of AI in veterinary practice.	[64]
Radiology	Images	DL, ResNet18 and 11 CAM explainability methods	Cats and dogs	7362	Among the evaluated CAM techniques, EigenGradCAM performed best; however, overall, the methods provided limited explainability and did not consistently enhance veterinarians’ diagnostic confidence across 9 pathologies.	[65]
Radiology	Images	DL, EfficientNet-B7 and Vision Transformer	Dogs	733 real and 1474 synthetic radiographs	With this model, analysis time was reduced and the Norberg angle was measured with higher accuracy than with the original method, except in cases of severe hip dysplasia.	[66]
Radiology	Images	DL, 3D U-Net	Dogs	221 canine CT scans	The model achieved high segmentation performance, underscoring the potential clinical applicability of this approach for liver segmentation in dogs.	[67]
Radiology	Images	DL, CNN	Dogs	Not specified (retrospective multicenter dataset)	The model achieved good accuracy in classifying different stages of myxomatous mitral valve disease from thoracic radiographs, demonstrating potential as a supportive tool for early diagnosis.	[68]
Radiology	Images	DL, CNN	Dogs	1465	The DL models showed high performance in detecting cardiomegaly from thoracic radiographs, supporting their use as computer-aided diagnostic tools in veterinary clinical settings.	[69]
Radiology	Images	ML, support vector machine	Dogs		The ML applied to MRI data identified key morphological features associated with Chiari-associated pain and syringomyelia, demonstrating strong diagnostic performance and potential for objective, data-driven assessment.	[70]

**Table 3:** Overview of recent studies (from 2020 to 2025) examining the application of AI in everyday care in companion animals by owners.

Field	Type of Data	AI Model	Species	Sample Size	Conclusion	Reference
Behaviour disorders monitoring	Images	DL, CNN	Dogs	Single dog, case report	An DL-enabled device supporting behavioral intervention significantly reduced separation anxiety signs, highlighting the potential of AI-assisted tools in managing behavioral disorders.	[77]
Health management	Images	DL, faster R-CNN + Mask R-CNN	Dogs	525 images	This study proposes a DL-based method for detecting animals’ key parts, generating skeletons, and recognizing motion without sensors, achieving up to 100% accuracy in action recognition.	[78]
Health management	None	None, letter paper	Companion animals	No sample	This work recognizes that AI chatbots like ChatGPT have great potential to support animal health care. Still, their safe and effective use requires informed owners, clear regulations, and close collaboration with licensed veterinarians.	[79]
Nutrition management	Images	DL, YOLOv5 detection network	Dogs	20,000 images of 120 dog breeds	This study demonstrated that the automatic pet feeder successfully meets its objectives by providing a reliable and adaptable solution for pet nutrition management.	[80]
Safety and tracking	Images	DL, contrastive learning, vision transformer	Dogs	78,702 real images	This paper introduces a conceptual framework for a prospective web application to help users locate missing pets. The application aims to improve the accuracy and efficiency of the search process.	[81]

**Table 5:** Overview of recent studies (2020–2025) examining the application of AI in monitoring companion animals’ behavior.

Type of Data	Dataset	AI Model	Species	Conclusion	Reference
Audio recordings	Sequencing data (GBS, MeDIP)	DL, CNN	Dogs	The DL models demonstrated high performance in classifying canine vocalizations, supporting automated approaches for behavioral monitoring and analysis.	[113]
Images	86,000 video frames (6000 in the test set)	DL, CNN	Dogs	Even at an early stage, the BlyzerDS system for quantifying sleep duration and fragmentation demonstrated strong accuracy in assessing dogs’ sleep behaviour. With comparable efficiency to manual observations, an autonomous behaviour analysis system could reduce the challenges of manually processing large video datasets, which is often slow, laborious, and prone to errors.	[114]
Images	Video recordings	DL, Mask R-CNN	Hamster	A DL–based video monitoring system successfully detected normal and abnormal behaviours, demonstrating potential for continuous animal monitoring and early anomaly detection.	[115]
Tabular	C-BARQ, ‘Stranger Test’ protocol data and video	ML, DL, Faster R-CNN, t-SNE clustering, multivariate logistic regression	Dogs	This study introduced an ML model to predict expert scores in a dog’s stranger test,’ achieving over 78% accuracy. The approach shows promise for digitally enhancing behavioural assessments, with future work focusing on larger datasets, additional protocols, and test–retest reliability.	[116]
Time series	Accelerometer, activity matched by video recordings, 12 cats	ML, random forest classifier and SOM network	Cats	This study created accelerometer-based models to classify cat behaviour, showing similar performance for collars and harnesses. While rare behaviours need larger samples for better accuracy, findings revealed colony cats were largely inactive, highlighting the potential of accelerometers and ML for health monitoring.	[117]
Time series	Multimodal sensor data, 10 cats	DL, LSTM	Cats	This applied DL long short-term memory (LSTM) model with accelerometer, gyroscope, and magnetometer data to detect cat activity, offering a highly accurate wearable-based approach to support feline welfare.	[118]
Time series	Accelerometers and gyroscopes	ML, several classifiers	Dogs	This study demonstrates that interpretable ML can reliably recognize a wide range of dog behaviours with high accuracy and highlight the potential of explainable, non-invasive monitoring systems to support veterinary care, training, and animal welfare.	[119]
Time series	Accelerometer, gyroscope, and magnetometer data	DL, CNN and LSTM hybrid model	Cats and dogs	The model demonstrates high accuracy in recognizing common activities of cats and dogs, achieving 89% accuracy for cats and 94% for dogs. These results confirm its applicability across diverse settings and highlight its potential for advancing automated behaviour monitoring and intelligent systems in animal–computer interaction and animal welfare research.	[120]
Time series	Accelerometer and gyroscope sensors	ML, several methods, Gaussian Naive Bayes with best results	Dogs	The ML models using wearable sensor data accurately classified canine activities, with Gaussian Naïve Bayes achieving the highest performance, supporting the use of sensor-based monitoring for assessing dog behaviour and welfare.	[121]
Time series	Video recordings	DL, K9-Blyzer software, based on Faster R-CNN with ResNet101 network	Dogs	Computational analysis of movement patterns enabled objective assessment of ADHD-like behaviour, supporting the use of AI tools for quantitative behavioral evaluation in clinical settings.	[122]
Time series	Video recordings	DL, FilterNet architecture (combined CNN and LSTM)	>2500 Dogs	A deep learning–based wearable system accurately classified a wide range of canine behaviours in real-world conditions, demonstrating strong potential for continuous health monitoring and early detection of behavioral changes.	[123]
Time series	Accelerometer and gyroscope sensors	ML, several classifiers	Dogs	The ML models fusing wearable sensor data accurately classified multiple canine activities, demonstrating the potential of sensor-based systems for behavioral monitoring and welfare assessment.	[124]
Videos	230 videos in 50 fps, 14 dogs	Ensemble Landmark Detector and statistical signal analysis	Brachycephalic and normocephalic dogs	This study demonstrates the value of the applied methodology in providing novel insights into communication with distinct patterns of facial expressivity between the two morphological groups of dogs.	[125]

## Data Availability

Not applicable.

## Supplementary Materials

The following supporting information can be downloaded at: https://www.mdpi.com/article/10.3390/ani16071035/s1, Supplementary Material S1: PRISMA 2020 Checklist. Reference [150] is cited in the supplementary materials.

## Abbreviations

AI	Artificial intelligence
ML	Machine learning
DL	Deep learning
NLP	Natural language processing
RGB	Read-green-blue color system
CNN	Convolutional neural network
LSTM	Long short-term memory
SOM	Self-organising map
t-SNE	t-distributed stochastic neighbor embedding
R-CNN	Region-based convolutional neural network
Fe-BARQ	Feline behavioural assessment and research questionnaire
MBTI	Myers–Briggs type indicator
BFI	Big five inventory
VOC	Volatile organic compound
CBC	Complete blood count
CAM	Class activation mapping
MLP	Multilayer perceptron
CT	Computed tomography
